# On correlations in IMRT planning aims

**DOI:** 10.1120/jacmp.v17i6.6411

**Published:** 2016-11-08

**Authors:** Arkajyoti Roy, Indra J. Das, Omid Nohadani

**Affiliations:** ^1^ Department of Industrial Engineering and Management Sciences Northwestern University Evanston IL USA; ^2^ Department of Applied Statistics and Operations Research Bowling Green State University Bowling Green OH USA; ^3^ Department of Radiation Oncology New York University Langone Medical Center New York NY USA

**Keywords:** IMRT plan evaluation, correlations, planning aims, statistical analysis, DVH

## Abstract

The purpose was to study correlations amongst IMRT DVH evaluation points and how their relaxation impacts the overall plan. 100 head‐and‐neck cancer cases, using the Eclipse treatment planning system with the same protocol, are statistically analyzed for PTV, brainstem, and spinal cord. To measure variations amongst the plans, we use (i) interquartile range (IQR) of volume as a function of dose, (ii) interquartile range of dose as a function of volume, and (iii) dose falloff. To determine correlations for institutional and ICRU goals, conditional probabilities and medians are computed. We observe that most plans exceed the median PTV dose (average $D_{50}\;=\;104\%$ prescribed dose). Furthermore, satisfying $D_{50}$ reduced the probability of also satisfying $D_{98}$, constituting a negative correlation of these goals. On the other hand, satisfying $D_{50}$ increased the probability of satisfying $D_{2}$, suggesting a positive correlation. A positive correlation is also observed between the PTV $V_{105}$ and $V_{110}$. Similarly, a positive correlation between the brainstem $V_{45}$ and $V_{50}$ is measured by an increase in the conditional median of $V_{45}$, when $V_{50}$ is violated. Despite the imposed institutional and international recommendations, significant variations amongst DVH points can occur. Even though DVH aims are evaluated independently, sizable correlations amongst them are possible, indicating that some goals cannot be satisfied concurrently, calling for unbiased plan criteria.

PACS number(s): 87.55.dk, 87.53.Bn, 87.55.Qr, 87.55.de.

## I. INTRODUCTION

Treatment plan optimization in intensity‐modulated radiation therapy (IMRT) often leads to unsatisfactory solutions due to constraints imposed to achieve clinical goals. To overcome infeasibilities, constraints are relaxed and competing objectives between tumor volume(s) and surrounding structures are formed. Competing objectives lead to trade‐offs for clinical use that are visually inspected and approved. The subjectivity of a visual inspection for IMRT planning decisions introduces biases and tendencies, consequently resulting in plans to become nonreproducible. In fact, Das et al.^(1)^ observed high variability among treatment planners and institutions. They reported that the median dose delivered to the tumor varied between ± 10% of the prescribed dose for 96% of the patient population. Any tendencies in treatment planning also lead to plan degeneracy, where multiple competing plans are produced, all satisfying institutional criteria (typically 95% volume receiving 95% dose to target). Even though all these guidelines are typically met, the significant personal preferences in plans raise concerns for effective and reproducible methods for comparing treatment plans among patients and institutions.

In general, such preferences may be introduced from a variety of sources, including delineation of anatomical structures, treatment planning system (TPS) variability, and institutional guidelines, but most importantly treatment planner's preferences. Accurately delineating tumors and organs is heavily dependent on both the quality of CT images and the experience of clinicians. Cazzaniga et al.^(2)^ compared structure delineation for three patients by six radiologists and observed standard deviations ranging from 0.35 to 2.64 cm^2^ of overlapping area. This reveals the need for more detailed constraints in addition to clinical experience in order to restrict such preferences. Fogliata et al.^(3)^ observed tendencies introduced through the choice of TPS by comparing eight TPSs on four patients using scoring indices. They reported varying scores for organs at risk (OAR) and target depending on the employed TSP. Eriguchi et al.^(4)^ observed variations due to institutional protocols by comparing treatment plans executed at multiple institutions using the same TPS on the same patient scans. They reported a $D_{50}$ to PTV that ranged from 43.6 to 51.2 Gy, and the volume that received 20 Gy or higher, $V_{20}$, ranged from 12.2% to 18.9% over the four institutions. $D_{x}$ is the minimum dose to the hottest x% of the volume and $V_{x}$ is the fractional volume that receives at least x% of the prescription dose.

In principle, clinical dose and volume constraints and guidelines are used to overcome the aforementioned sources of variations, as well as to assess the quality of plans.^5^, ^6^ However, despite rigorously imposing these constraints, Das et al.^(7)^ observed substantial differences in the DVH shapes when varying patients, planners, and TPS. An additional factor is the allocation of time to generate a plan, as typically very little time is devoted for optimization.^(8)^ Dosimetric uncertainties can also alter the quality of plans.^(9)^

The DVH constraints are often imposed through specific dose‐volume points as a comparison metric, such as $D_{2}$, $D_{50}$, $D_{95}$, and $D_{98}$. They may also directly serve as DVH constraints in plan optimization, as suggested by Cambria et al.^(5)^ In order to adhere to these constraints, treatment plans are often iteratively adjusted by relaxing one or more constraints to achieve the desired result. This iterative process is heavily dependent on the planner's skill and experience, as demonstrated by Nelms et al.,^(10)^ who suggested the need for best‐practices criteria as part of a continuous improvement strategy to reduce significant plan variations. Subjective preferences in trading off DVH constraints during the planning are reflected in the final plan's DVH control points and their correlations.

In this work, we study the dependence amongst these DVH control points. Since we cannot assume the DVH variations amongst cases to follow a Gaussian distribution, nonparametric statistical tools are employed in two main steps:

1. Variations amongst plans, measured by median and interquartile range.
2. Correlations between planning aims, computed via conditional probability and median.

We observe significant variations surrounding the DVH constraints even though most of the clinical goals were satisfied. It is shown that the current clinical DVH constraints require prioritizing amongst them, which limits the reproducibility and standardized plan quality. Furthermore, we demonstrate trends in increased dose delivery, suggesting dissension in evaluating treatment plans. The details of materials used in the study are discussed in the Materials & Methods section. Three statistical measures are detailed in subsection A.1 to demonstrate the variation in dose, volume, and DVH falloff along the DVH. To show the efficacy of clinical goals, statistical comparisons between plans that violate and those that satisfy the constraints are presented in subsection B.

## II. MATERIALS AND METHODS

One hundred head‐and‐neck cancer cases from Indiana University, Melvin and Bren Simon Cancer Center are selected based on their anatomical comparability. The corresponding treatments for all cases are planned using the same TPS (Eclipse, Varian Medical System, Palo Alto, CA), following identical institutional dosimetric criteria. Besides anatomical differences, possible variations stem from plans that were produced by different planners. Based on clinical advice, brainstem and spinal cord are identified as the highest priority OAR to be spared. For brevity, we shall refer to spinal cord as spine. In many cases when the lesion was at or adjacent to the skin, the patient was scanned with bolus. Consequently, the PTV is deliniated to be within 2–3 mm of the skin surface.^(11)^ For the cases with multiple PTVs, the one with the largest prescribed dose is identified as the primary target to be irradiated. We analyze all targets and report the primaries as the main PTV for this study to maximize the contrast between the competing objectives of tumor irradiation and OAR sparing.

These plans were optimized based on dosimetric constraints for PTV of $D_{95}\;\geq \;95\%$ and $D_{2}\;\leq \;107\%$ of the prescribed dose, for spine $D^{\text{max}}\;\leq \;45\;\text{Gy}$, and for brainstem $D^{\text{max}}\;\leq \;54\;\text{Gy}$, following the institutional guidelines. All DVHs are collected from the TPS with a resolution of 0.1 cGy dose. Furthermore, the DVHs for PTV are collected using relative volume and dose points, allowing effective comparisons. Note that the median absolute prescribed dose over all patients is 70 Gy, with an interquartile range of 10.3 Gy. Moreover, in order to compare specific dose‐volume points, DVH values are linearly extrapolated by binning remaining doses with 0% volume.

The quantitative analysis is divided into two parts: variations amongst plans and correlations between DVH control points. For the first part, we use three statistical metrics that are well suited for such nonsymmetrically distributed data, namely the interquartile range of dose for changing volume, interquartile range of volume for changing dose, and the approximate DVH gradient to study variation in the falloff. For the second part, we use conditional measures for institutional and for internationally recommended DVH goals. Note that the methods and the results section will follow this two‐part structure. The details of our analytical method are as follows.

### A. Variations in plans

The interquartile range is an estimator of spread, computed as the difference of the upper and the lower quartile $\left(Q_{3}{‐Q}_{1}\right)$ of a distribution. It is inherently robust to changes in scale, hence uninfluenced by outliers.^(12)^ Robustness is important, because an otherwise optimal solution (statistical estimator) may render suboptimal and unreliable when uncertainties occur.^13^, ^14^ To study the spectrum of relevant variations amongst plans, we employ: (i) the IQR of volume as a function of dose, (ii) the IQR of dose as a function of volume, and (iii) the median DVH falloff and its IQR. The details are as follows.

#### A.1 Interquartile range of volume

${\text{IQR}}^{\text{vol}}\left(D\right)$ is a statistical estimator of $V_{x}$ distribution at a dose value D and quantifies directly the spread. In this study, ${\text{IQR}}^{\text{vol}}\left(D\right)$ is recorded over the entire dose range in increments of 0.1% relative dose (tumor) or 0.1Gy (OAR).

#### A.2 Interquartile range of dose

${\text{IQR}}^{\text{dose}}\left(V\right)$ measures the spread of $D_{x}$ distribution and is recorded over the entire volume range at V = 0.1% increments. Note that ${\text{IQR}}^{\text{dose}}\left(V\right)$ and ${\text{IQR}}^{\text{vol}}\left(D\right)$ are estimators of two different and orthogonal distributions and, hence, independent of each other.

#### A.3 Dose falloff

∇ serves as a measure for the DVH falloff. It is determined at two neighboring DVH point pairs (D1, V1) and (D2, V2) via finite differences

**Figure 1:**
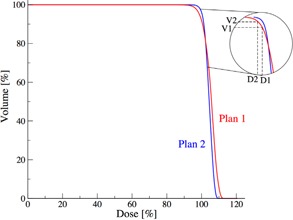
Schematic of two plans with a magnification of the crossing points.

∇=(V2−V1)/(D2−D1)

where D2‐D1 = 0.1%. *V2* and *V1* are the corresponding volumes. The schematic setting is illustrated in Fig. 1. The objective of this analysis is to identify whether a rapid falloff or a slow falloff (blue or red DVH in Fig. 1) was preferred for a specific plan.

In order to observe such variations and identify trends amongst past decisions, we compute the median DVH falloff, ∇(D), for all patients as function of dose. Additionally, the interquartile range of the falloff, ${\text{IQR}}^{\nabla }$, is determined in order to observe potential dissension in decision‐making favoring a slow or rather a rapid DVH falloff.

### B. Correlations in plans

The iterative treatment planning process aims to satisfy DVH constraints determined by both institutional and internationally recommended aims. In practice, however, it is often not feasible to satisfy all constraints, which in part justifies the existence of institutional guidelines which, in some cases, deviates from the recommended aims. As a result, treatment planners choose to trade off some constraints. The underlying assumption of this pursuit is that it is admissible to violate one or more of the aims, while still providing an overall satisfactory and clinically acceptable plan. To analyze the dependence amongst the constraints, we determine statistical correlations for PTV, brainstem, and spine constraints.

For each DVH control point i, we divide the set of the plans into those that satisfy the constraint i (subset $Y_{i}$) and those that violate it (subset $\bar{Y_{i}}$) with $Y_{i}\cap {\bar{Y}}_{i}=\emptyset$. To establish correlations, we use conditioning for two differing control points i and j as

$$P(Y_{i}|Y_{j})\geq P(Y_{i}|{\bar{Y}}_{j})\to positive\;correlation,$$

$$P(Y_{i}|Y_{j})<P(Y_{i}|{\bar{Y}}_{j})\to negative\;correlation,$$

where P(⋅|⋅) denotes the conditional probability. In other words, if violating the constraint j reduces the probability of constraint i to be met, these two constraints are positively correlated and negative otherwise. Here, probability reflects the relative number of plans in the subset.

Additionally, we quantify the rate of change by computing conditional medians and derive conclusions analogous to Eqs. (2) and (3).

In addition to these imposed goals, we also consider empirical control points to measure the correlations of the imposed goals to their surrounding DVH sections. To this end, when the plans exhibit unanimous agreement at a particular high dose by displaying a marginal spread, defined as ${\text{IQR}}^{\text{vol}}\left(D\right)\;\leq \;5\%$, we denote the corresponding (constrained) dose as $D^{\text{con}}$. Conversely, neighboring unconstrained DVH points (within ± 5%) with significant deviation (i.e., ${IQR}^{vol}(D)\gg 5\%$) are denoted as $D^{\text{unc}}$ (unconstrained dose). Such constrained and unconstrained points subsequently serve to qualify additional subsets. The analysis is divided for DVH constraints that stem from institutional protocols and those recommended by the ICRU‐83.^(15)^ The control points are as follows

#### B.1 Institutional aims

The institutional dosimetric constraints for PTV are $D_{95}\;\geq \;95\%$ and $D_{2}\;\leq \;107\%$ of the prescribed dose, for spine $D^{\text{max}}\;\leq \;45\;\text{Gy}$, and for brainstem $D^{\text{max}}\;\leq \;54\;\text{Gy}$. These control points are used to establish subsets of satisfying or violating plans, along with their neighboring points. The corresponding $D^{\text{con}}$ and $D^{\text{unc}}$ are data‐specific and will be discussed in the Results section below.

#### B.2 ICRU‐83 aims

ICRU guidelines recommend for head‐and‐neck cases, near‐max dose $D_{2}\;\leq \;107\%$, median dose $D_{50}\;=\;100\%$, and near‐min dose $D_{98}\;\geq \;95\%$ constraints for PTV, and $D^{\text{max}}$ for the surrounding OARs.^(15)^ In order to sizably distinguish violations from satisfying the $D_{50}$ goal, we extend the definition to encompass $D_{50}\;=\;100\%\;\pm \;2\%$.

## III. RESULTS

**Figure 2:**
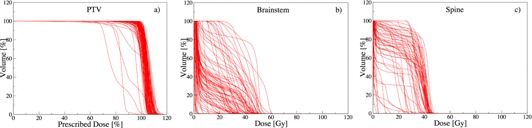
Dose‐volume histograms of 100 head‐and‐neck cancer patients.

Despite consistent planning protocols and the normalizing nature of DVH, sizable variations amongst the 100 head‐and‐neck cases are observed for PTV, brainstem, and spine in Figs. 2(a), (b), and (c), respectively. Maximum PTV dose ranges 104% and 120% of the prescribed dose. While the DVHs for most cases overlap for PTV, we observe a wider spread for brainstem and spine (see Figs. 2(a) to (c)). For brainstem, a rapid decrease is apparent in low dose areas and a plateau in the mid‐volume range for most cases. For spine, we observe an extended plateau at high‐volume range with a decrease for higher dose for most cases. To quantify these variations, we employ the estimators introduced in Material and Methods section 7.

### A. Variation estimators

As observed in Figs. 2(a) to (c), the distribution amongst plans is not symmetric. This is expected, given the nonincreasing nature of cumulative DVH. Therefore, IQR is well suited to characterize the spread of the asymmetrical DVH distributions. We consider an IQR > 5% at a DVH goal to indicate a significant variation amongst plans.

#### A.1 Interquartile range of volume

**Figure 3:**
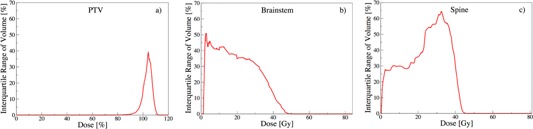
Interquartile range of volume as a function of dose: ${\text{IQR}}^{\text{vol}}\left(D\right)$.

In clinical practice, volume constraints are often imposed at specific points, to achieve desired dose distributions (e.g., $V_{95}$ on tumor).^(16)^ In order to meet these DVH constraints, the weights on other clinical objectives are adjusted and at times completely relaxed. These decisions are case‐specific and depend on the discretion of the oncologist and/or planner, leading to variations of $V_{x}$ over the patients. The spread of this distribution can be measured by ${\text{IQR}}^{\text{vol}}\left(D\right)$ and is illustrated in Fig. 3.

Figure 3(a) shows unanimous agreement of PTV's ${\text{IQR}}^{\text{vol}}\left(D\right)$ until 90% prescribed dose $\left({\text{IQR}}^{\text{vol}}\;=\;0\%\right)$. The maximum deviation is reached at ${\text{IQR}}^{\text{vol}}\left(104\right)\;=\;38\%$, and vanishes again at ${\text{IQR}}^{\text{vol}}\left(111\right)\;=\;0\%$, depicting the DVH falloff region. The observed peak at D = 104% indicates an increased spread at D = 104%, which can stem from relaxed PTV constraints or how the objectives are specified, amongst others. Note that the recommendations of the Radiation Therapy Oncology Group allow for up to 20% of the tumor volume to receive more than 110% prescribed dose, which supports the observed tendency to tumor excess dose.^(17)^ The overall narrower and lower ${\text{IQR}}^{\text{vol}}\left(D\right)$ of PTV reflects the higher importance assigned to PTV in comparison to brainstem and spine.

Figure 3(b) illustrates that the volume variation for brainstem peaks at ${\text{IQR}}^{\text{vol}}\left(4\right)\;=\;51\%$, followed by a second peak at ${\text{IQR}}^{\text{vol}}\left(17\right)\;=\;43\%$ with a subsequent steady decrease and vanishes at ${\text{IQR}}^{\text{vol}}\left(50\right)\;=\;0\%$. One possible explanation would be that treatment planners prefer to relax constraints on brainstem at lower dose values, but tend to much tighter control at higher dose values, supported by the observed narrower spread. For spine, Fig. 3(c) reveals an increase of ${\text{IQR}}^{\text{vol}}\left(10\right)\;=\;30\%$ to ${\text{IQR}}^{\text{vol}}\left(32\right)\;=\;65\%$, followed by a sharp decrease to ${\text{IQR}}^{\text{vol}}\left(45\right)\;=\;0\%$. This result indicates tight constraints on higher doses for spine, whereas the large spread for D = [20,40] Gy suggests relaxation. However, a tighter control is not necessary because the probability of myelopathy vanishes for D < 40 Gy to the cord.^(18)^ These observations may be considered as an indirect effect of the predominant $D^{\text{max}}$ constraints on brainstems and spines, as common in head‐and‐neck cases.^19^, ^20^, ^21^, ^22^ Note that $D^{\text{max}}$ thresholds lead to V = 0% at $D\;\geq \;D^{\text{max}}$, resulting in narrower ${\text{IQR}}^{\text{vol}}$.

#### A.2 Interquartile range of dose

**Figure 4:**
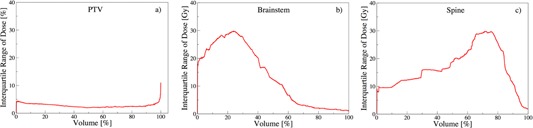
Interquartile range of dose as a function of volume: IQR^dose^(V).

In addition to the volume constraints, a set of constraints are imposed at specific dose values. Similarly, they may lead to variations in the surrounding DVH regions which we measure with ${\text{IQR}}^{\text{dose}}\left(V\right)$. For PTV, Fig. 4(a) illustrates negligible $\left({\text{IQR}}^{\text{dose}}\;=\;3\%\right)$ for V ≤ 99%, with a steep increase to ${\text{IQR}}^{\text{dose}}\left(100\right)\;=\;11\%$. This suggests that tight $D_{x}$ constraints are imposed on PTVs until V ≤ 95% followed by relaxed control for higher volumes, which is in agreement with the recommendations.^(23)^

Figure 4(b) shows reduced ${\text{IQR}}^{\text{dose}}$ at higher brainstem volumes. This is in agreement with the recommendations that strict $D^{\text{max}}$ constraints are essential to maintain functionality of such critical organs.^19^, ^20^, ^21^, ^22^ However, Fig. 4(c) for spine depicts significant spread around $D_{60}{‐D}_{80}$, indicating a lack of $D_{50}$ constraint and a need for tighter control at higher doses.

#### A.3 Dose falloff

**Figure 5:**
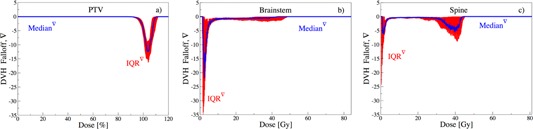
DVH falloff: blue line marks the median ∇(D) and red the interquartile range ${\text{IQR}}^{\nabla }$.

To achieve the goal of conformal target dose, intensity modulation is leveraged to deliver heterogeneous distributions, allowing for a sharp dose falloff on DVHs.^(24)^ A rapid dose falloff at the prescribed dose allows to reduce risks of complications.^(25)^ A vertical falloff at the prescribed dose constitutes an ideal target DVH. To achieve this goal, near‐minimum dose $\left(D_{98}\right)$ and near‐maximum dose $\left(D_{2}\right)$ constraints are imposed.^(26)^ However, due to competing objectives, a sharp and perpendicular falloff is usually infeasible. Therefore, some constraints are relaxed, leading to V variations amongst plans. The median ∇ and ${\text{IQR}}^{\nabla }$ are shown in Figs. 5(a) to (c) for PTV, brainstem, and spine.

Figures 5(a) to (c) show a marginal planning variation for PTV, in comparison to brainstem and spine. This is seen by narrow ${\text{IQR}}^{\nabla }$ surrounding the prescribed dose, which is typically a result of employing multiple constraints. For brainstem and spine, however, the significant ${\text{IQR}}^{\nabla }$ is spread over multiple regions, demonstrating variabilities in decision‐making as a result of employing single maximum dose constraints. More specifically, Fig. 5(a) shows a vanishing PTV median ∇(D) for D ≤ 95%, followed by a drop to median ∇(104) = ‐13. This implies that a dose change of 1% results in a ‐13% volume change. In other words, the falloff region V = [100,0]% extends over a median dose interval of 7.7%(= 100/13), exhibiting a narrow falloff region through tighter control. This is followed by a rise to ∇(110) = 0. This change in median ∇(D) for D = [95,110]% coincides with the region surrounding the prescribed dose. Noticeably, the sharpest falloff occurs at D = 104% instead of D = 100%, further suggesting planners’ preference towards higher dose, as also reported by Eisbruch et al.^(17)^

Figure 5(b) illustrates that the sharpest falloff for brainstem occurs at very low‐dose values (D = 2 Gy), demonstrating tight dose constraints. Unlike brainstem, the median ∇(D) for spine in Fig. 5(c) shows two distinct turning points at D = 2 Gy and at D = 40 Gy. The first DVH falloff is attributed to an artifact of the patient geometry (for some cases, lower doses to significant portions of organs is common, but not for all). On the other hand, the turning point at D = 40 Gy is a direct result of maximum dose constraints for brainstem and spine, which are typically imposed in head‐and‐neck cancer cases.^19^, ^20^, ^21^, ^22^

The spread around the median is shown via ${\text{IQR}}^{\nabla }$ in Figs 5(a) to (c). For PTV, negligible spread is observed for D ≤ 90%, followed by narrow spread for D = [90,95]% region, which is typically a result of strict constraints.^(23)^ However, significant spread occurs around the DVH falloff region, revealing variability in decision‐making over this range, followed by the overall agreement beyond D ≥ 110%. For brainstem, the largest ${\text{IQR}}^{\nabla }$ is recorded at lower dose values, followed by a narrow deviation at higher dose points. This further suggests multiple constraints imposed at higher dose for this critical organ. Similarly for spine, beyond the low‐dose spread, a significant spread is observable only between D = 30 Gy and D = 45 Gy. This demonstrates both the impact of maximum dose constraints and a lack of median dose constraints. Since the maximum dose constraints are typically rigorously enforced on both PTV and critical organs, competing dose‐volume constraints are relaxed to generate feasible plans.

Note that the use of statistical estimators for symmetric distributions would misrepresent the actual distribution here. For example, the use of mean and standard deviation for ∇ reveals a substantial ripple at $D_{85}$ (shown in Appendix A), which is not observable with median ∇ and ${\text{IQR}}^{\nabla }$ in Fig. 5(a). However, this ripple is caused by four outliers, while the median and interquartile ranges are insensitive to outliers and, hence, appropriately describe the observed asymmetric distribution. While such outliers may reveal additional insights, their analysis is beyond the scope of this work.

### B. Correlations in plans

Since all the studied plans were clinically approved, it is justified to expect broad agreement at dose‐volume constraints enforced in the study, while variations may exist at other constraints which were relaxed. To establish possible consequences of such goal relaxation, we included empirical constraints in the vicinity of the DVH goal points. This allows for a guide to gauge potential risks for the overall plan that are associated with violating some constraints. We first focused the analysis on the institutional DVH goals that were enforced for the studied cases. We then analyze the cases based on additional constraints recommended by the ICRU‐83,^(15)^ since a subset of them overlaps with the institutional constraints.

#### B.1 Institutional aims

When the plans exhibit unanimous agreement at a particular high dose by displaying a marginal spread (i.e., ${\text{IQR}}^{\text{vol}}\left(D\right)\;\leq \;5\%$), we denote the corresponding (constrained) dose as $D^{\text{con}}$. We observed a PTV constrained dose point with ${\text{IQR}}^{\text{vol}}\left(D\right)\;\leq \;5\%$ at $D^{\text{con}}\;=\;110\%$. For brainstem, $D^{\text{con}}\;=\;50\;\text{Gy}$ and for spine, $D^{\text{con}}\;=\;45\;\text{Gy}$. This corresponds to maximum dose constraints that are usually imposed on all structures, often being the only constraint for brainstem or spine.^19^, ^20^, ^21^, ^22^ When $V_{110}$, $V_{50}$, or $V_{45}$ are negligible (≤ 2%), we hypothesize them to be a consequence of maximum dose constraints, and otherwise (> 2%) independent. On the other hand, we observe a neighboring (within ± 5%) unconstrained point with ${IQR}^{vol}(D)\gg 5\%$ at $D^{\text{unc}}\;=\;105\%$ for PTV, $D^{\text{unc}}\;=\;45\;\text{Gy}$ for brainstem, and $D^{\text{unc}}\;=\;40\;\text{Gy}$ for spine. Note that, due to the nonincreasing nature of cumulative DVHs, $V_{105}\;\geq \;V_{110}$, $V_{45}\;\geq \;V_{50}$, and $V_{40}\;\geq \;V_{45}$ for PTV, brainstem, and spine, respectively. This implies that an equal or larger spread (from V ≤ 2% objective) is expected at $D^{\text{unc}}$ when compared to $D^{\text{con}}$, since the constraints are found at higher volumes. To establish deviations, we observed two clusters of plans, namely those satisfying $V_{105}$, $V_{45}$, or $V_{40}$ by being less or equal to 2% and those which were significantly different at a neighboring (± 5%) point $D^{\text{unc}}$. The two groups maximally separate, when the volumes at $D^{\text{unc}}$ exceeded 45%. Therefore, the hypothesis is that such violations (> 45%) are related to meeting other DVH constraints, and otherwise (≤ 45%) independent. To summarize these relationships, Table 1 serves as an overview on institutional planning aims. Note that each table element can serve as a subset, as defined in Materials and Methods section B.

**Figure 6:**
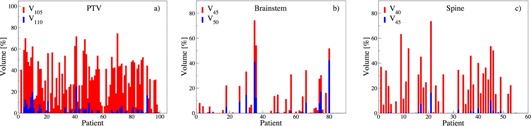
Impact of DVH volume constraints. Large volume deviations are observed at $D^{\text{unc}}$ (red) compared to $D^{\text{con}}$ (blue).

Significant overdose tendencies are observed for PTV. For brainstem, minimal deviations in comparison to spine is apparent, which has higher percentage of constraint violations. This demonstrates the increased planning importance for brainstem. Specifically, Fig. 6(a) for PTV shows substantial violations at $V_{105}$ (> 65% for some patients) in comparison to $V_{110}$, where violations are negligible (on average ≤ 2%). This implies substantial volume portions received D > 100%, supporting aforementioned observation of tendencies to deliver higher doses to PTV. More importantly, this result shows larger violations at $D^{\text{unc}}$ for cases that also have slight violations at $D^{\text{con}}$. This result is more evident when evaluating the conditional median. In particular, the conditional median of $V_{105}$ given a violation at $V_{110}$ is Median$\left(V_{105}\;|\;V_{110}\;>\;2\%\right)\;=\;50\%$ compared to Median$\left(V_{105}\;|\;V_{110}\;\leq \;2\%\right)\;=\;19\%$ for PTV.

**Table 1:** Summary of planning aims: left) when constrained DVH points are violated or satisfied; right) when significant deviations or independent points are observed

		*Constrained Dose* $\left(D^{\text{con}}\right)$		*Neighboring Dose* $\left(D^{\text{unc}}\right)$	
*Structure*	*Planning Aims*	*Criteria Violated (%)*	*Criteria Satisfied (%)*	*Significant Deviation (%)*	*Independent of D* ^*con*^ *(%)*
PTV	$V_{95}$	$V_{95}\;<\;95$	$V_{95}\;\geq \;95$	$V_{100}\;<\;45$	$V_{100}\;\geq \;45$
	$D^{\text{max}}$	$V_{110}\;>\;2$	$V_{110}\;\leq \;2$	$V_{105}\;>\;45$	$V_{105}\;\leq \;45$
Brainstem	$D^{\text{max}}$	$V_{50}\;>\;2$	$V_{50}\;\leq \;2$	$V_{45}\;>\;45$	$V_{45}\;\leq \;45$
Spine	$D^{\text{max}}$	$V_{45}\;>\;2$	$V_{45}\;\leq \;2$	$V_{40}\;>\;45$	$V_{40}\;\leq \;45$
		*Constrained volume* ($V^{con}$)		*Neighboring volume* ($V^{unc}$)	
PTV	$D_{95}$	$D_{95}\;<\;95$	$D_{95}\;\geq \;95$	$D_{100}\;<\;70$	$D_{100}\;\geq \;70$

Similarly, $D_{95}\;=\;95\%$ constraints (denoted as $V^{\text{con}}$) were imposed on PTV, which is a common institutional choice.^(27)^ The neighboring volumes are considered $V^{\text{unc}}$ (e.g., at $D_{100}$). Patients with significant deviations (D < 70%) at $D_{100}$ also show deviations from the established $D_{95}$ constraints (not shown). The conditional Median$\left(D_{110}\;|\;D_{95}\;<\;95\%\right)\;=\;62\%$ versus Median$\left(D_{110}\;|\;D_{95}\;\geq \;95\%\right)\;=\;90\%$ shows improvement in neighboring areas when constraints are satisfied.

Figure 6(b) illustrates negligible disagreement at $D^{\text{unc}}$ for brainstem. Even though violations are observed, they are not due to decision tendencies but rather due to the nonincreasing nature of cumulative DVHs and the already existing violations at $D^{\text{con}}$. When comparing the conditional medians for brainstem, we observe a difference between Median$\left(V_{45}\;|\;V_{50}\;>\;2\%\right)\;=\;33\%$ and Median$\left(V_{45}\;|\;V_{50}\;\leq \;2\%\right)\;=\;0\%$. For spine, Fig. 6(c) shows significant disagreement at $D^{\text{unc}}$ propagated with violations at $D^{\text{con}}$. There is also a sizable difference between Median$\left(V_{45}\;|\;V_{50}\;>\;2\%\right)\;=\;45\%$ and Median($\left(V_{45}\;|\;V_{50}\;\leq \;2\%\right)\;=\;9\%$ for spine. This result indicates that marginal deviations from DVH constraints can (in median) lead to significant degradation in neighboring unconstrained points and, hence, limit the plan quality. Overall, Fig. 6 shows the case‐by‐case impact of constraint violation for all cases. Beyond the conditional changes, we next provide a quantitative guide for the associated risk for a plan when constraints are violated.

In practice, not all clinical goals can be satisfied concurrently, resulting in constraint relaxation. To measure the risk (probability) that violating some constraints may have on other goals, we evaluate the plans at $D^{\text{con}}\;=\;110\%$ for PTV and $D^{\text{con}}\;=\;50\;\text{Gy}$, and 45 Gy for brainstem, and spine, respectively. We hypothesize that $D^{\text{con}}$ satisfaction is correlated to $D^{\text{max}}$ constraints, or otherwise (> 2%) independent. Additionally for PTV, we evaluate $D^{\text{con}}\;=\;95\%$, for which we hypothesize that $V_{95}\;\geq \;95\%$ if satisfied, or $V_{95}\;<\;95\%$ if violated. For correlation analysis, we divide all plans into subsets defined in Table 1 and apply the conditional measures of Materials and Methods section B:

Subset A: plans satisfying $D^{\text{max}}$ goal, leading to V ≤ 2% at $D^{\text{con}}$;

Subset B: plans satisfying $V_{95}$ goal, leading to V ≥ 95% at $D^{\text{con}}$;

Subset $\bar{C}$: plans violating low volume at high‐dose goal, leading to V ≥ 45% at $D^{\text{unc}}$.

**Figure 7:**
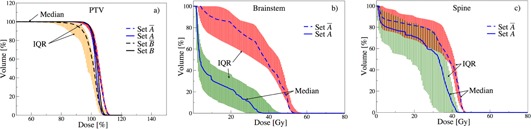
DVH for subsets of plans: A satisfy the $D^{\text{max}}$ constraint and B meet $V_{95}$ goal.

Figure 7 summarizes the DVHs of plans belonging to the subset A (B) or its compliment $\overset{\&}{A}\left(\overset{\&}{B}\right)$. For PTV, $V_{110}$ violation (subset $\bar{A}$) leads to negligible excess dose. However, $V_{95}$ violation (subset $\bar{B}$) has a greater impact through significant underdose, as observed by the broader IQR that is asymmetric towards lower volumes and dose. Note that the IQR limits depict plans that significantly degrade due to $V_{95}$ violation $\left(\overset{\&macr;}{B}\right)$.

For brainstem, a substantial improvement is observed for plans that satisfy $V_{50}$ (subset A). In fact, the IQR of A and $\bar{A}$ do not overlap, suggesting that significant improvements are possible, if the constraints are satisfied. For spine, moderate reduction in dose is observed for plans that satisfy the $V_{45}$ constraint (subset A), suggesting a similar potential for improvements. Figure 7 shows that violating constraints goes beyond the common notion of “trade‐off” and actually affects the entire DVH. It allows one to determine the impact of violating one of these constraints on the overall plan quality. Therefore, this analysis can inform clinical planning decisions to quantitatively gauge potential risks of constraint relaxations.

This impact can also be accessed by comparing the probabilities of delivering high doses to larger volume portions in neighboring and unconstrained DVH points $\left(D^{\text{unc}}\right)$ when conditioned on violating (or meeting) the goals at $D^{\text{con}}$. In other words, we compare the conditional probability $P(\bar{C}–|\bar{A}–)(\text{at}\;D^{\text{con}})\;\text{to}\;P(\bar{C}–|A)\;(\text{at Dcon})$). Therefore, the impact of not meeting PTV's constraints is observed through the comparison of

$$P_{PTV}(\bar{C}|\bar{A})=P(V_{105}>45\%|V_{110}>2\%)=\frac{20/100}{31/100}=0.65$$

and

$$P_{PTV}(\bar{C}|\bar{A})=P(V_{105}>45\%|V_{110}\leq 2\%)=\frac{6/100}{69/100}=0.09.$$

This demonstrates a positive correlation between $V_{105}$ and $V_{110}$ for the PTV, namely violating one increases the probability of violating the other as well.

Similarly for brainstem, the probabilities for degrading the plans are $P_{\text{brainstem}}\left(\overset{\&macr;}{C}\;|\;\overset{\&macr;}{A}\right)\;=\;0.33$ and for spine $P_{\text{spine}}\left(\overset{\&macr;}{C}\;|\;\overset{\&macr;}{A}\right)\;=\;0.60$. In comparison when $D^{\text{con}}$ are met, $P_{\text{brainstem}}\left(\overset{\&macr;}{C}\;|\;A\right)\;=\;0$ and $P_{\text{spine}}\left(\overset{\&macr;}{C}\;|\;A\right)\;=\;0.08$. The two proportion z‐tests, $P\left(\overset{\&macr;}{C}\;|\;\overset{\&macr;}{A}\right)$ reveals significantly larger than $P\left(\overset{\&macr;}{C}\;|\;A\right)$ for all structures with a p‐value less than 0.0001.

Additionally for PTV, $P\left(D_{100}\;<\;70\%\;|\;D_{95}\;<\;95\%\right)\;=\;0.69$ is significantly larger than $P\left(D_{100}\;<\;70\%\;|\;D_{95}\;\geq \;95\%\right)\;=\;0.01$. Furthermore, the two proportions z‐tests confirm this significant difference (p‐value < 0.0001). Therefore, all these observations support the conclusion that significantly higher risks for degrading outcomes are expected when DVH constraints are violated. These results can serve to quantitatively inform the decision‐making process in treatment planning.

#### B.2 ICRU aims

Similarly, we analyze the correlations amongst the noninstitutional aims, as defined in section B of Material and Methods. Table 2 summarizes the relevant internationally recommended planning aims by ICRU‐83.^(15)^

These aims may also become competing (i.e., they cannot be met concurrently), hence, compelling planners to relax or even ignore some of them. To measure correlations among the ICRU constraints, we similarly group patients into subsets as discussed in Materials and Methods section B:

Subset E: plans that satisfy $D_{2}\;\leq \;107\%$;

Subset F: plans that satisfy $98\%\;\leq \;D_{50}\;\leq \;102\%$;

Subset G: plans that satisfy $D_{98}\;\geq \;95\%$.

**Figure 8:**
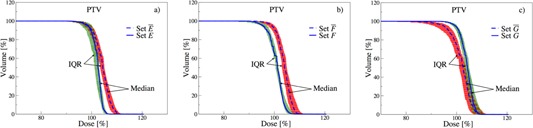
Analysis of the ICRU‐83 recommendations for PTV: (a) $D_{2}$ satisfied (E) or violated $\left(\overset{\&macr;}{E}\right)$, (b) $D_{50}$ satisfied (F) or violated $\left(\overset{\&macr;}{F}\right)$, (c) $D_{98}$ satisfied (G) or violated $\left(\overset{\&macr;}{G}\right)$.

The impact of violating the ICRU‐83 recommendations is shown in Fig. 8. The excess dose violation of subset $\bar{E}$ and the underdose violation of subset $\bar{G}$ in Fig. 8(a) can be considered as a result of the definition of these constraints. For the subset $\bar{F}$ in Fig. 8(b), the $D_{50}$ violation is attributed to an increased dose, confirming the overall overdose tendency observed through this analysis. This is also reflected in an elevated dose to larger volumes, hence satisfying $D_{98}$, as demonstrated in Fig. 8(c) for the subset G. Conversely for F, $D_{50}$ is satisfied by reduced dose to larger volumes, which leads to violating $D_{98}$ and constituting $\bar{G}$. In other words, plans in subset F have an increased likelihood to also reside in $\bar{G}$. Similarly, those in $\bar{F}$ are more probable to also be in G. Therefore, the set of analyzed plans clearly suggests a negative correlation between the recommended aims of $D_{50}$ and $D_{98}$, indicating competing goals that are unlikely to be met simultaneously.

The pairwise conditional probabilities for all goals are summarized in Table 3. Given that 86% of the cases violate $D_{50}$ (subset $\bar{F}$), the probabilities of F conditioned on E or G are marginal. However, when conditioning on F, a sizable positive correlation is observed for the subset E, since the corresponding probability decreases upon complementing F. In other words, these plans suggest that satisfying $D_{50}$ increases the probability of satisfying $D_{2}$ as well. On the other hand, a negative correlation is observed when G is conditioned on F, since the probability increases when conditioning on $\bar{F}$. This means that satisfying $D_{50}$ reduces the probability of satisfying $D_{98}$. This observed competing nature of these two recommended aims demonstrates that the likelihood of meeting them simultaneously is low.

**Table 2:** Summary of ICRU aims for PTV and the criteria for violation or satisfaction

*Planning Aims*	*Criteria Violated (%)*	*Criteria Satisfied (%)*
$D_{2}\;\leq \;107$	$D_{2}\;>\;107$	$D_{2}\;\leq \;107$
$D_{50}\;=\;100$	$98\;>\;D_{50}\;>\;102$	$98\;\leq \;D_{50}\;\leq \;102$
$D_{98}\;\geq \;95$	$D_{98}\;<\;95$	$D_{98}\;\geq \;95$

**Table 3:** Probabilities of subset $Y_{i}$ in row i is conditioned on $Y_{j}$ in column j. For sizable differences, a positive correlation is marked in blue and a negative correlation in red

*Subsets* $Y_{i}\backslash Y_{j}$	$P\left(Y_{i}\;|\;Y_{j}\right)$				
*E*	$\bar{E}$	*F*	$\bar{F}$	*G*	$\bar{G}$
E	0.31		**0.64**	**0.26**	0.30	0.32
$\bar{E}$		0.69	**0.36**	**0.74**	0.70	0.68
F	0.29	0	0.14		0.07	0
$\bar{F}$	0.71	1.0		0.86	0.93	1.07
G	0.68	0.70	**0.36**	**0.74**	0.69	
$\bar{G}$	0.32	0.30	**0.64**	**0.26**		0.31

Since these plans were designed and delivered to the best ability and knowledge of experts, the result that not all ICRU‐83 recommended aims could be satisfied supports the clinical need for institutional guidelines, such as $D_{95}$, as prevalent amongst institutions. At the same time, we observe that these institutional goals lead to trends, such as excess dose tendencies, that potentially jeopardize both plan quality and their comparability across institutions.

## IV. DISCUSSION

In treatment planning, point‐wise dose‐volume goals are not only used to enforce clinical objectives, or to directly optimize treatment plans, but also to choose between alternative plans. The analysis presented in this work seeks to identify areas of plan variation and constraint correlation. Therefore, a possible extension of the presented study includes developing and integrating unbiased constraints and estimators directly into treatment planning to mitigate variability.

To limit dissension in decision‐making, this study reveals the need for constraints or comparison estimators that can be enforced for extended DVH regions. The incorporation of an unbiased estimator for treatment plan evaluation was suggested by Loveless et al.^(28)^ A deviation metric was designed to capture the weighted difference between the realized and ideal DVH. The weights were extracted from past treated cases. Alternatively, benchmark DVHs were used for treatment comparisons for prostate cancer cases.^(29)^

It needs to be noted that this analysis was confined to head‐and‐neck cases only, warranting comparable anatomies. Unique geometries for some patients still may contribute to deviations in received dose on certain structures. These variations are patient‐specific and can occur in any treatment. Therefore, we chose robust estimators to immunize the conclusions against potential uncertainties. Furthermore, since the main goal of this study was to establish correlations, the resulting probabilities and risk measures are indented to inform future treatment planning, but cannot yield causations. The understanding of the nature of these effects, potentially confounded by the geometry, treatment planning system, dosimetric models, and other factors, is beyond the scope of this study. Nevertheless, these results have the potential to quantitatively augment the treatment planning, confining the trial‐and‐error nature of the decision‐making process and, hence, reducing time and ambiguity.

## V. CONCLUSIONS

The impact of trading off clinically employed DVH goals for treatment planning is demonstrated using three robust statistical estimators on 100 past head‐and‐neck cases. Significant plan deviations are observed on the interquartile ranges of dose and volume, as well as DVH falloff, especially on areas neighboring the constraints. This analysis shows that, when these constraints are even marginally violated, larger deviations are expected across the entire DVH. These results extend conventional notions of a “trade‐off” between clinical goals to a quantitative risk measure that relates each constraint violation to degradation of the overall plan. In fact, some of the internationally recommended aims were identified to be negatively correlated, hence, competing. This study also identifies DVH goals of unanimous agreement amongst planners, as well as areas of dissension and deviations, calling for novel clinical constraints that go beyond the established dose‐volume constraints and address extended areas of the DVH. Since these results relied on standard dosimetric criteria, they are of general nature and, hence, applicable to a wide range of IMRT planning settings.

## ACKNOWLEDGMENTS

We would like to thank S. Srivastava for technical support in data extraction and M. Langer for insightful discussions. O. Nohadani thanks the support from the National Science Foundation under Grant No. CMMI 1463489.

## COPYRIGHT

This work is licensed under a Creative Commons Attribution 3.0 Unported License.

## APPENDICES

### Apendix A: Supplementary Materials

#### 1. Conventional statistical estimators

**Figure A.1:**
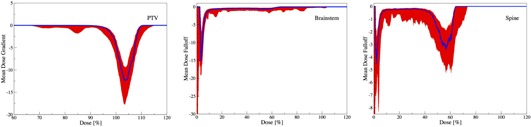
DVH falloff. The blue line marks the mean ∇(D), and the red line the standard deviation.

When the distribution of data is symmetric and in the absence of outliers, conventional statistical estimators, such as sample mean and standard deviation, provide reliable analytical tools for the observations. However, when the distribution is not symmetric and outliers are prevalent, this is no longer the case. Figure A.1 demonstrates the use of mean and standard deviation for the dose falloff ∇ of all three structures. The observed ripple at $D_{85}$ for PTV suggests an overall phenomenon for this control point. A further analysis of the data reveals that the standard deviation was impacted by only four outlier plans for which specific tumor geometries required deviating arrangements. This ripple, however, does not speak for the cohort of the plans. Similar ripples are observable for brainstem and spine, all representing nonglobal uncertainties. On the other hand, robust estimators, such as the median and IQR, are immune to these pertubations, as exhibited in Fig. 5 of the manuscript.

#### *2. Institutional* $D_{95}$ *control points*

**Figure A.2:**
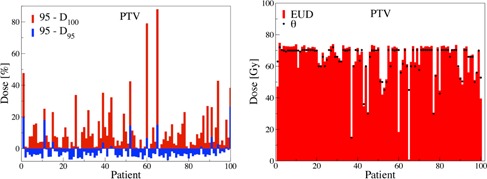
(left) $D_{95}$: large deviations at $D^{\text{unc}}$ (red) compared to $D^{\text{con}}$ (blue); (right) EUD to PTV for all patients. Black stars mark the prescribed dose Ø.

Figure A.2(left) shows that patients with sizable violations (D < 70%) at $D_{100}$ also show some deviations from the established $D_{95}$ constraint. This analysis demonstrates that when the institutional constraint at $D_{95}$ is relaxed, the dose point $D_{100}$ is sizably degraded for most cases. On the other hand, when $D_{95}$ is met, the value at $D_{100}$ is also satisfactory.

#### 3. Equivalent uniform dose

Equivalent uniform dose (EUD) is computed as $\text{EUD}={\left(\sum_{i}v_{i}D_{i}^{\alpha }\right)}^{1/\alpha }$, where $v_{i}$ is the i‐th partial volume receiving the dose $D_{i}$ and an exponent of α = ‐10 for the tumor.^(1)^ The EUD was computed for equal dose per fraction schedules based on the biologically effective dose method.^(1)^ Figure A.2(right) shows the EUD to PTV for all patients. For almost all plans, the EUDs are near the prescribed dose, showing negligible variations.

## Supporting information

Supplementary Material

Supplementary Material

Supplementary Material

Supplementary Material
