# Supplementary material for: On correlations in IMRT planning aims
Source: J Appl Clin Med Phys. 2016 Nov 8;17(6):44–59. doi: 10.1120/jacmp.v17i6.6411 (PMC5690524; doi:10.1120/jacmp.v17i6.6411)
Supplement: Supplementary file 2 — Supplementary Material [file ACM2-17-044-s002.doc]

##### Supplements to

##### “On Correlations in IMRT Planning Aims”

## 1 Conventional statistical estimators

When the distribution of data is symmetric and in the absence of outliers, conventional statistical estimators, such as sample mean and standard deviation, provide a reliable analytical tools for the observations. However, when the distribution is not symmetric and outlier are prevalent, this is not longer the case. Figure 1supp) demonstrates the use of mean and standard deviation for the dose falloff ∇ of all three structures. The observed ripple at *D*_85_ for PTV suggests an overall phenomenon for this conrol point. A further analysis of the data reveals that the standard deviation was impacted by only four outlier plans for which specific tumor geometries required deviating arrangements. This ripple, however, does not speak for the cohort of the plans. Similar ripples are observable for brainstem and spin, all representing non-global uncertainties. On the other hand, robust estimators, such as themedian and IQR, are immune to these pertubation, as exhibited in Figure 5 of the manuscript.


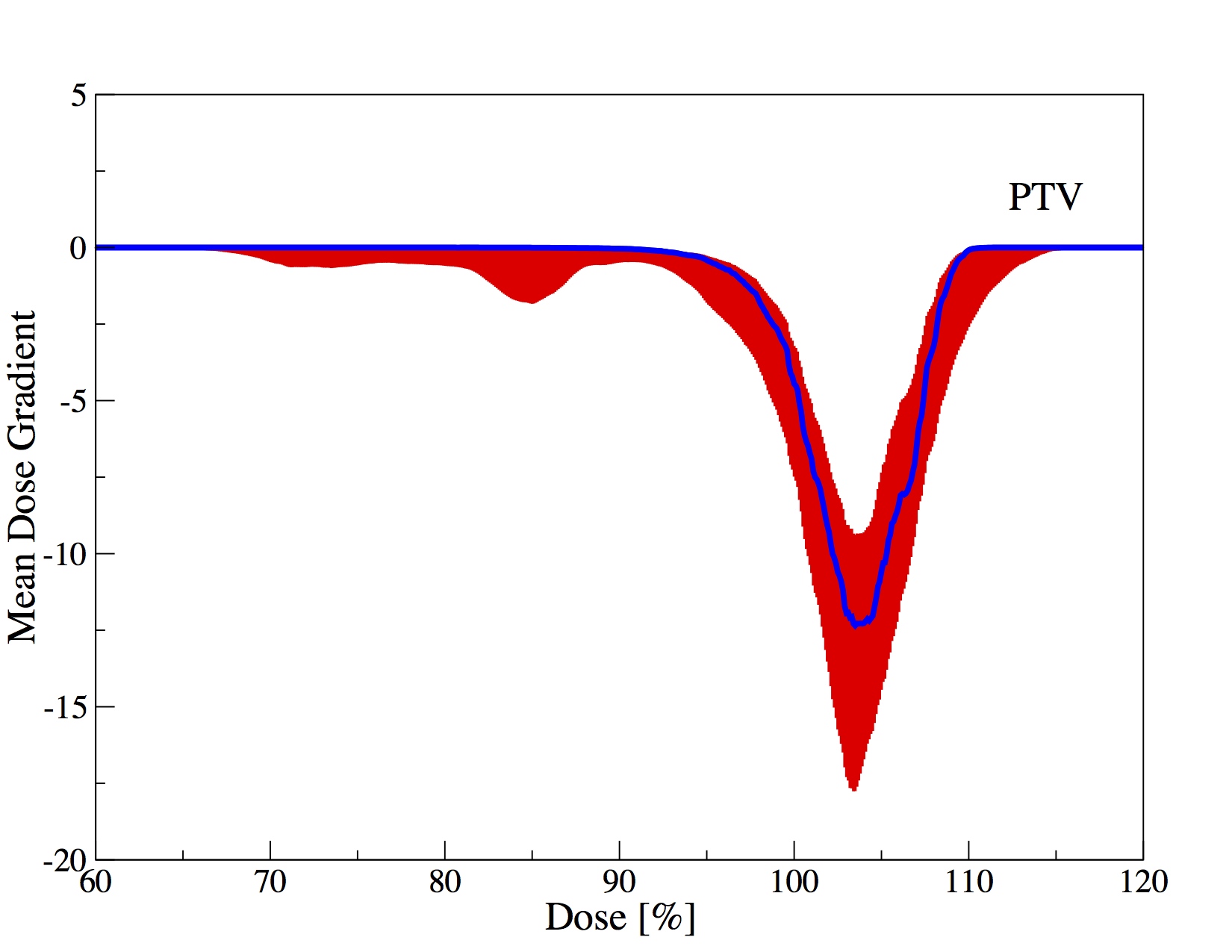

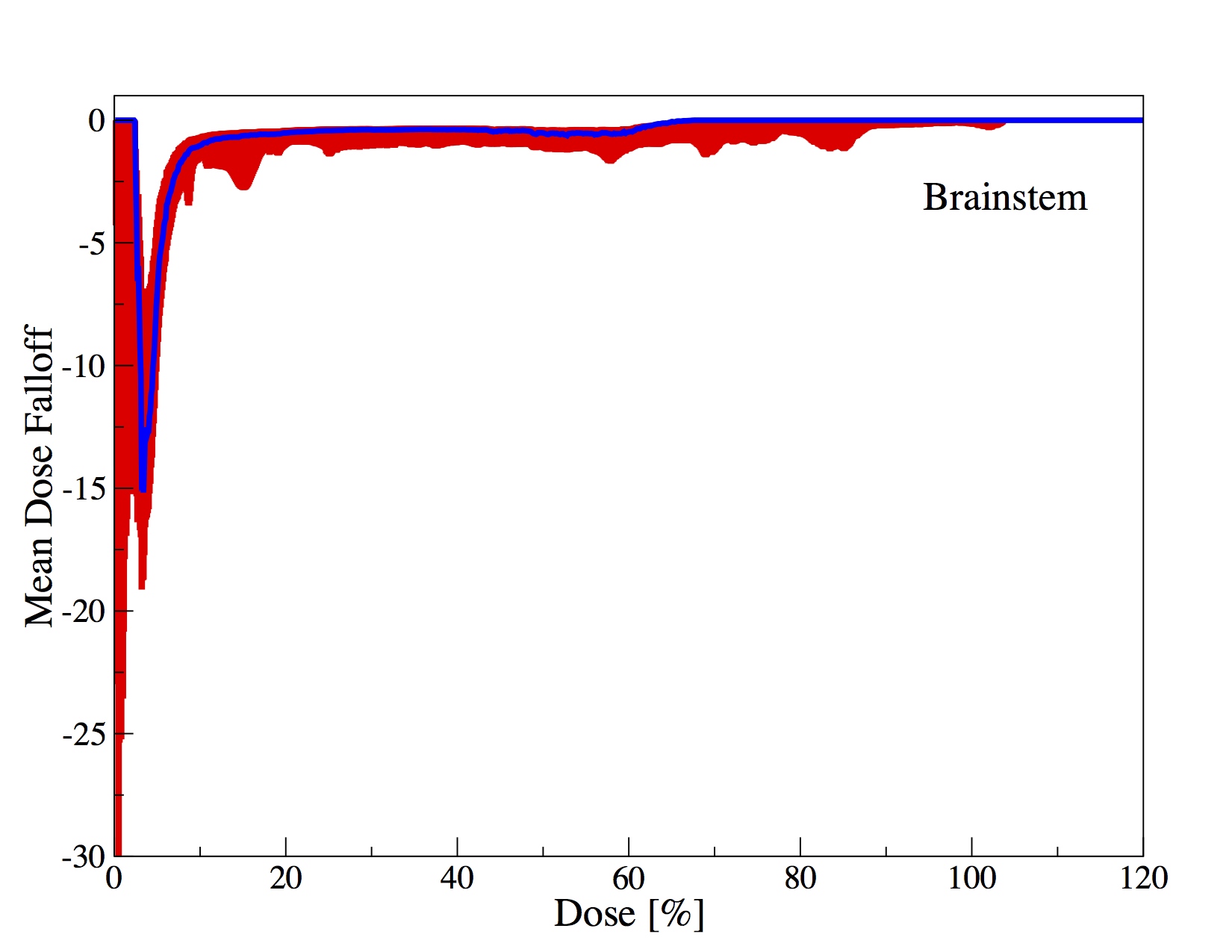

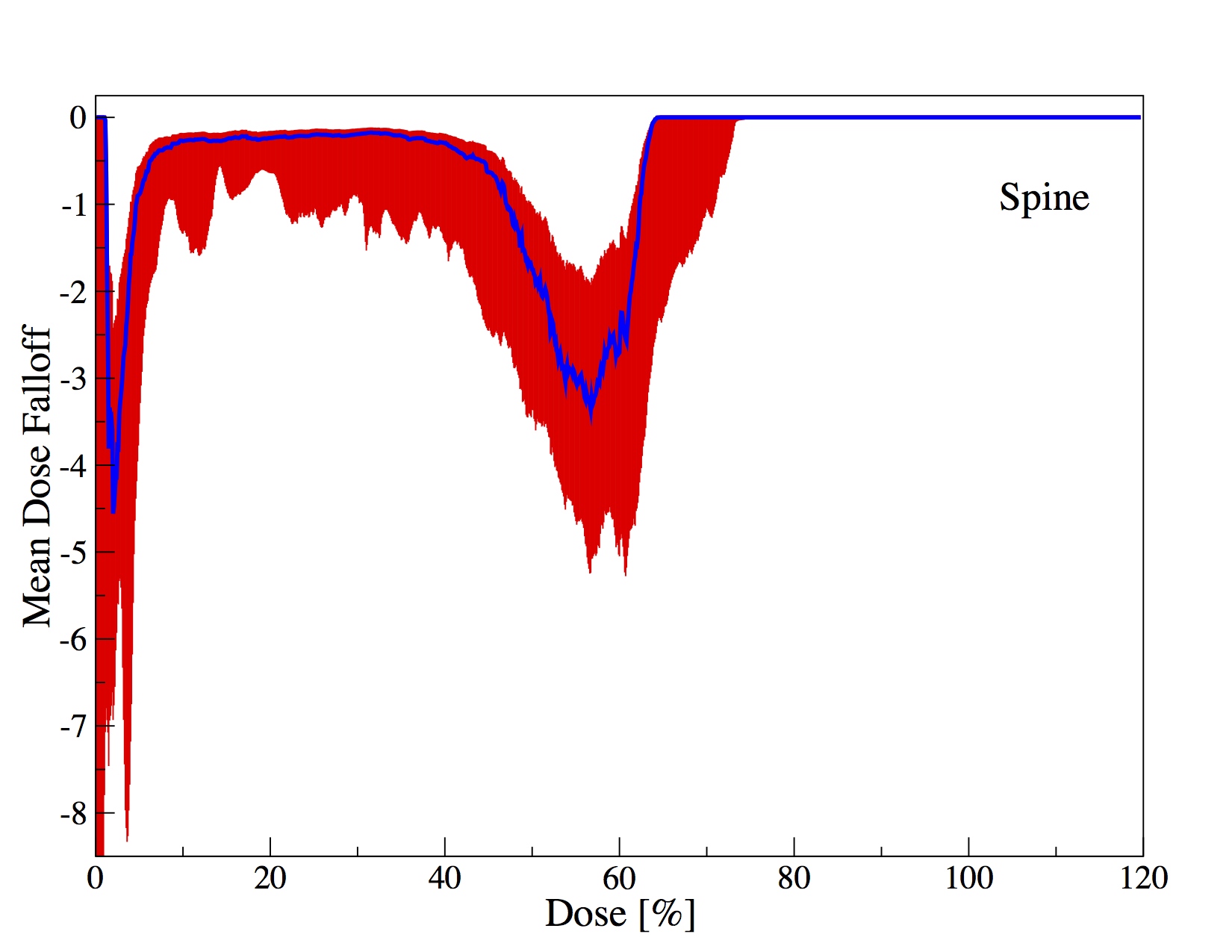


Figure 1supp: DVH falloff: blue line marks the mean ∇(*D*) and red the standard deviation.

## 2 Institutional *D_95_* control points

Figure 2supp-left) shows that patients with sizable violations (*D* < 70%) at *D_100_* also show some deviations from the established *D_95_* constraint. This analysis demonstrates that when the institutional constraint at *D_95_* is relaxed, the dose point *D_100_* is sizable degraded for most cases. On the other hand, when *D_95_* is met, the value at *D_100_* is also satisfactory.


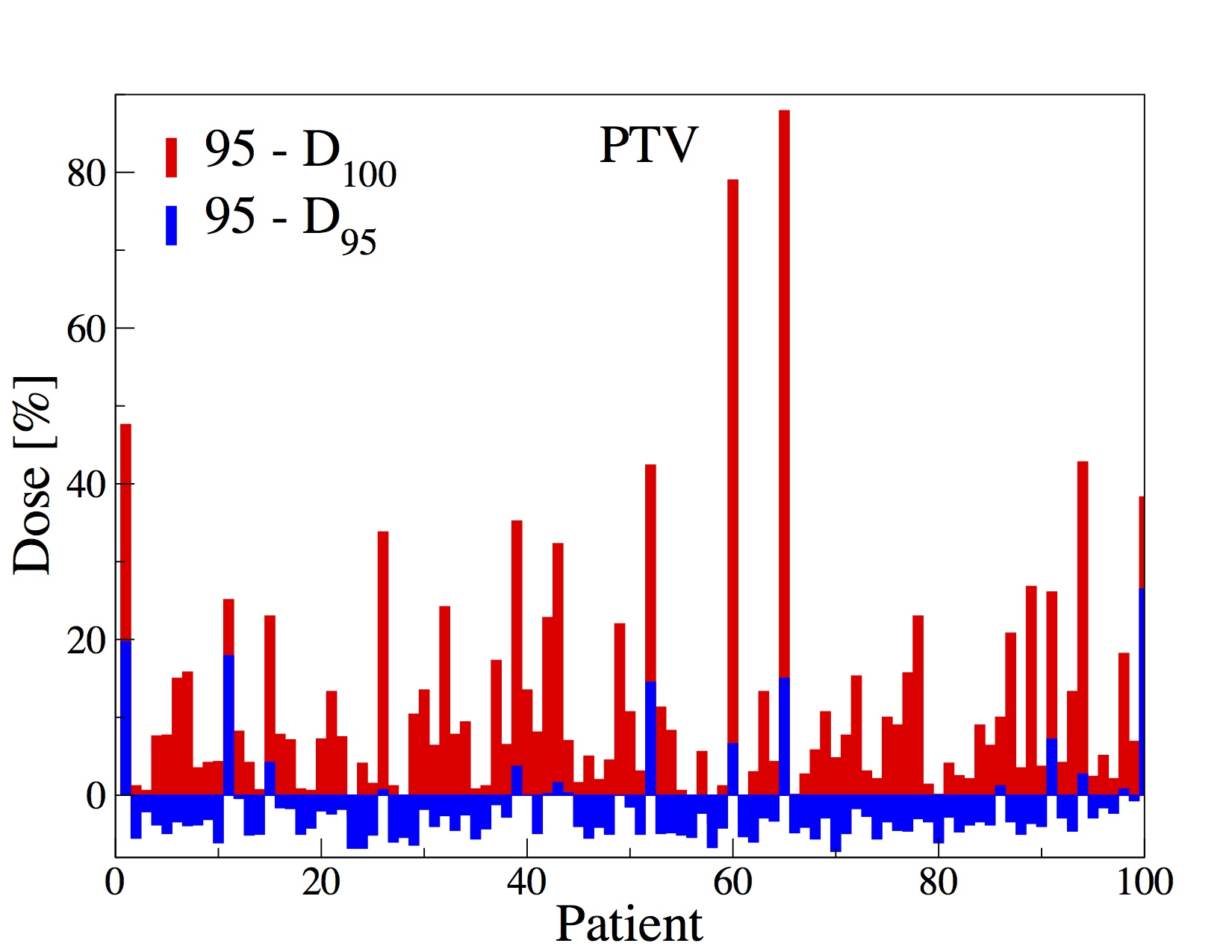


Figure 2supp: left) *D_95_*: large deviations at *D^unc^* (red) compared to *D^con^* (blue);

right) EUD to PTV for all patients. Black stars mark the prescribed dose θ.

## 3 Equivalent Uniform Dose

Equivalent uniform dose (EUD) is computed as EUD *= ( ∑ v_i_ D_i_^α^ )^1/α^*, where *v_i_* is the *i*-th partial volume receiving the dose *D_i_* and an exponent of *α* = −10 for the tumor.^(1)^ The EUD was computed for equal dose per fraction schedules based on the biologically effective dose method.^(1,2)^ Figure 2supp-right) shows the EUD to PTV for all patients. For almost all plans, the EUDs are near the prescribed dose, showing negligible variations.
